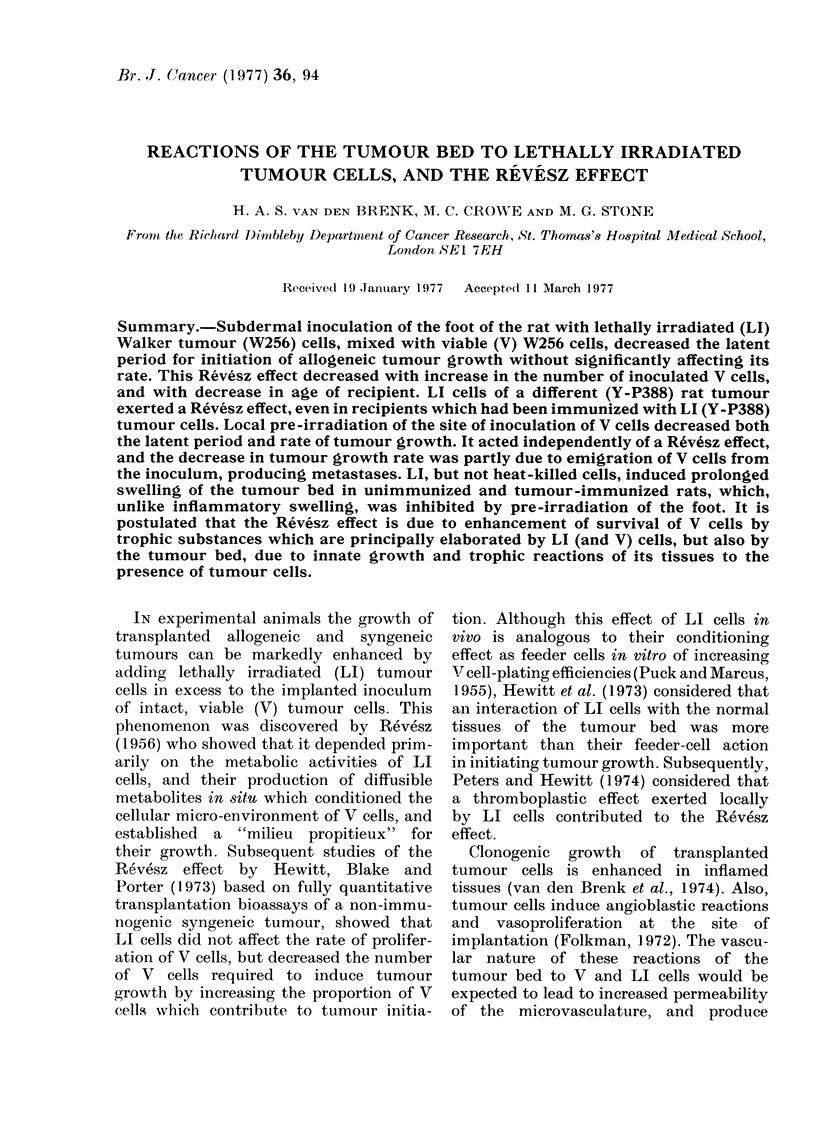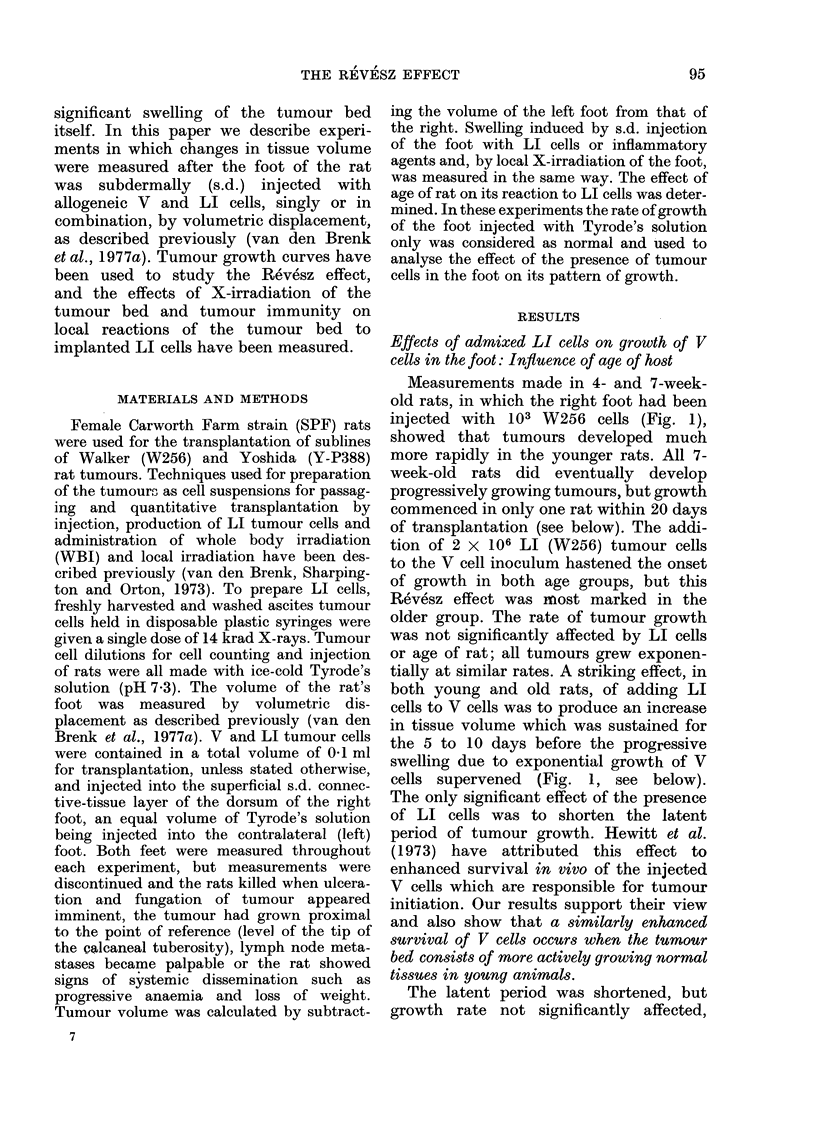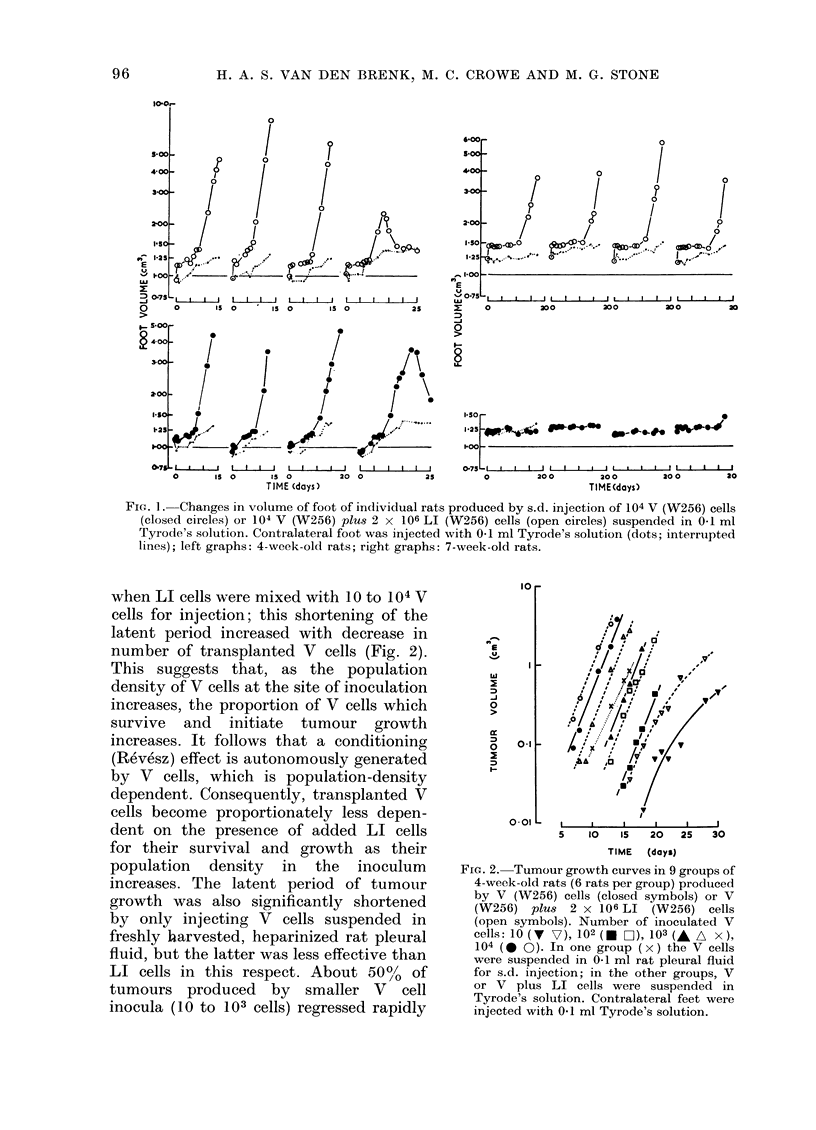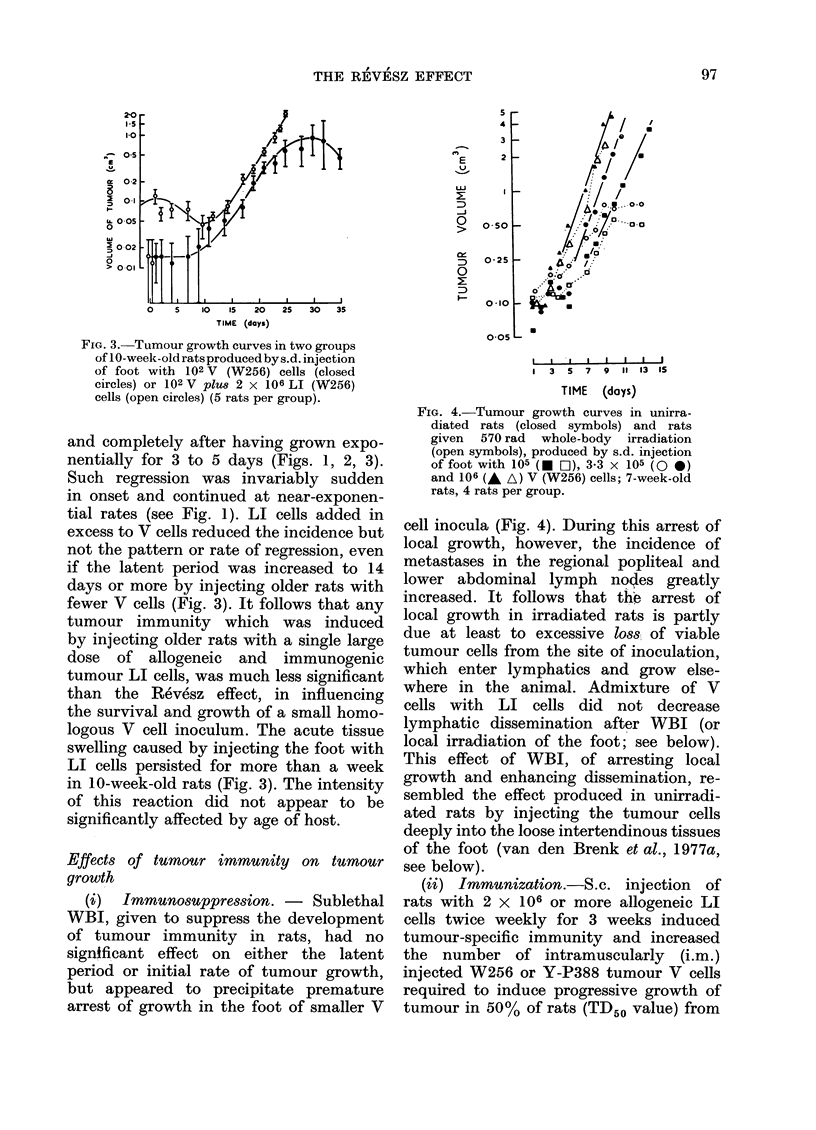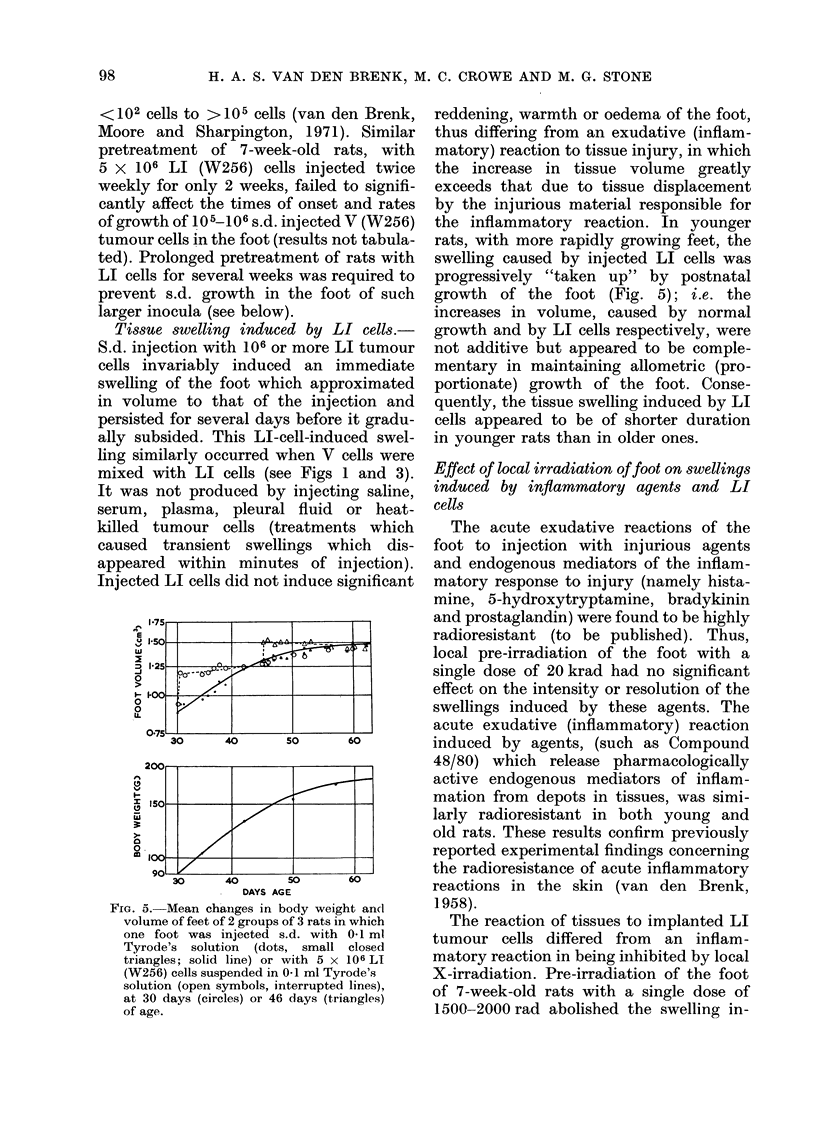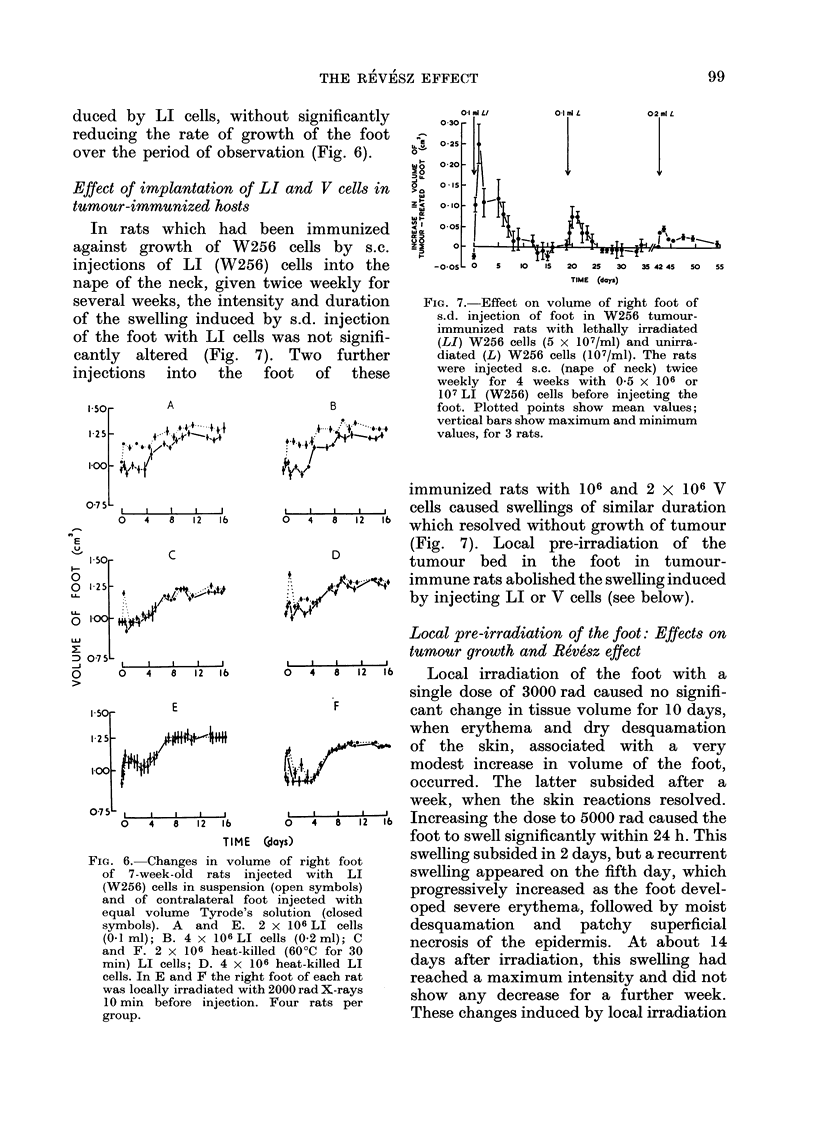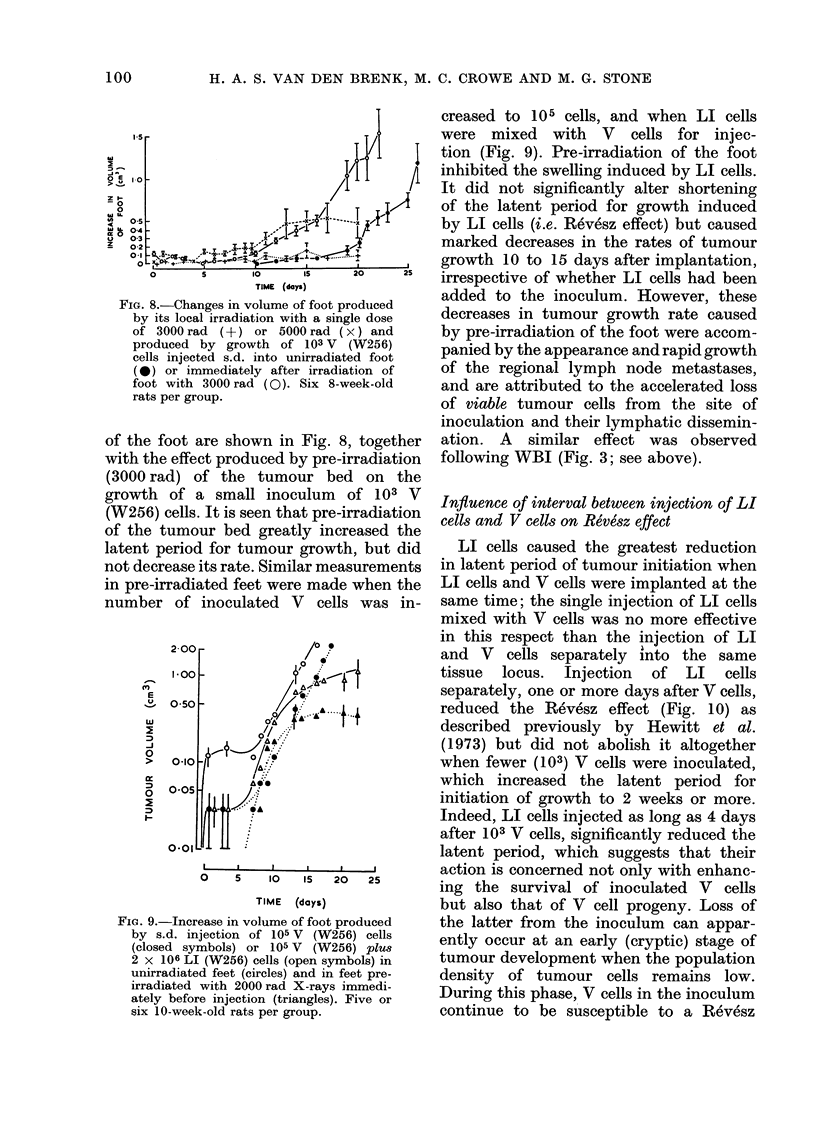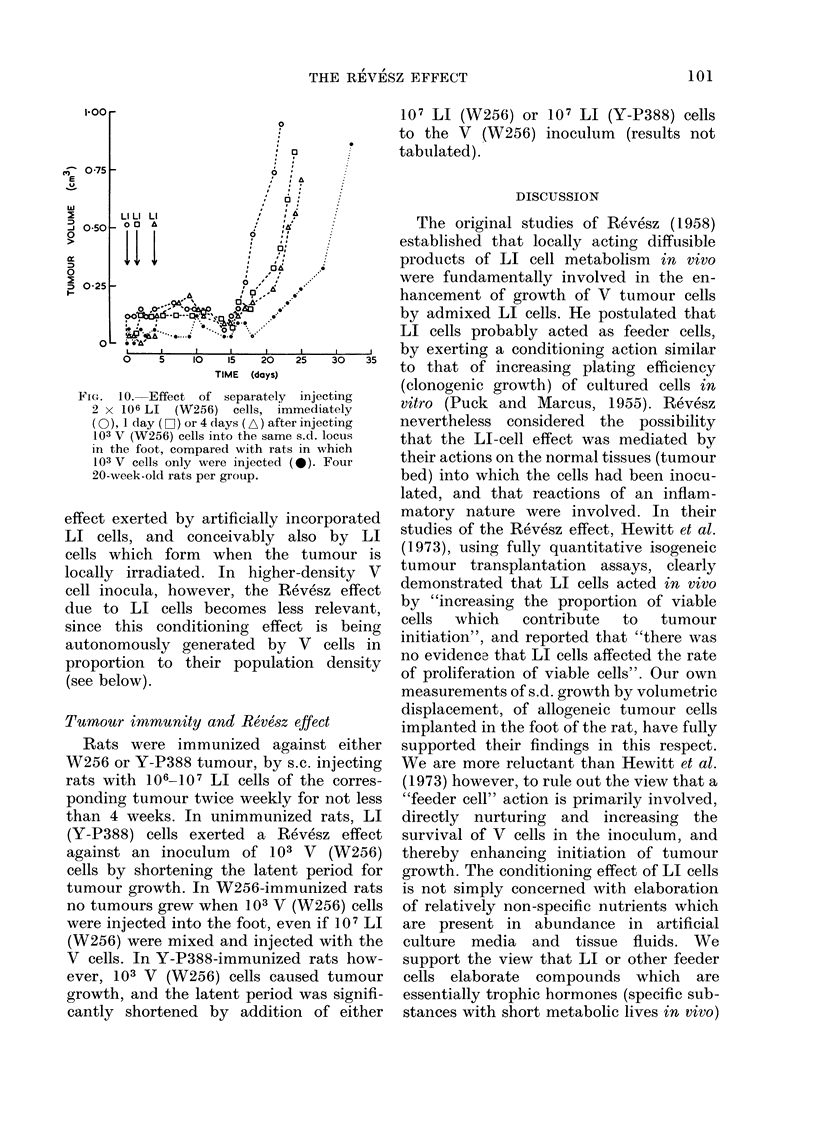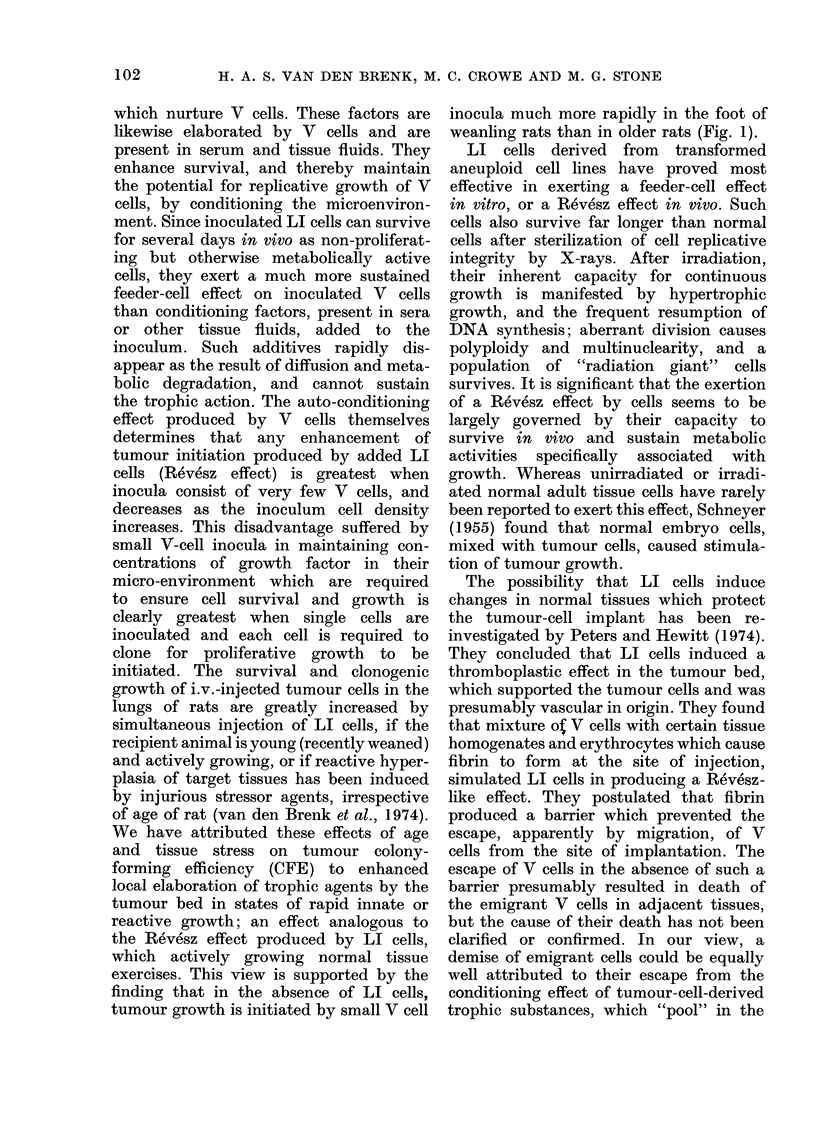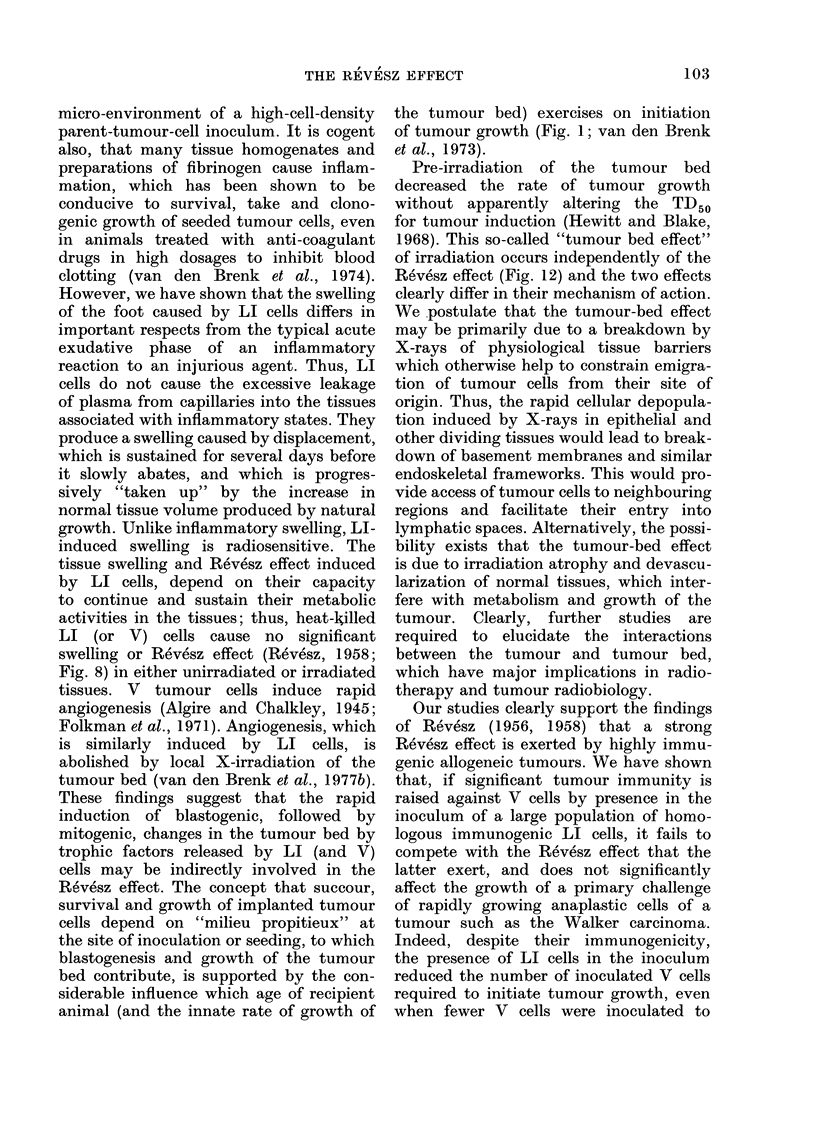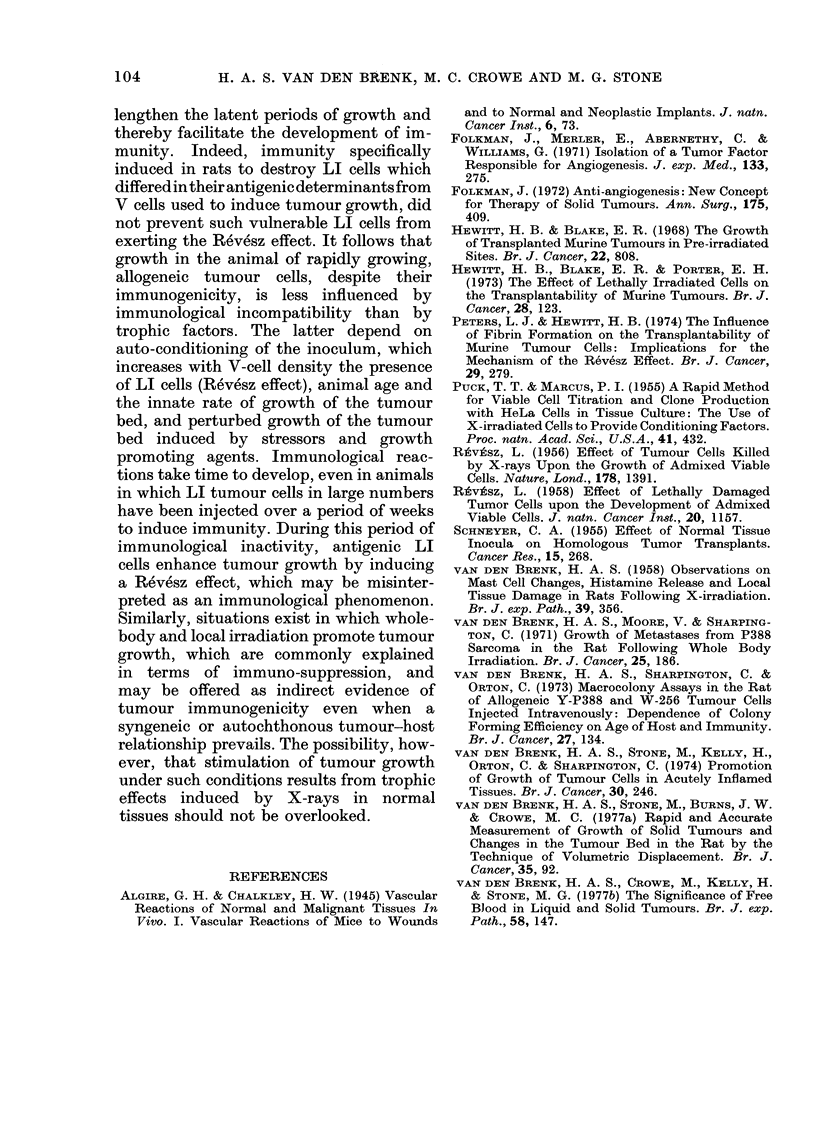# Reactions of the tumour bed to lethally irradiated tumour cells, and the Révész effect.

**DOI:** 10.1038/bjc.1977.159

**Published:** 1977-07

**Authors:** H. A. van den Brenk, M. C. Crowe, M. G. Stone

## Abstract

Subdermal inoculation of the foot of the rat with lethally irradiated (LI) Walker tumour (W256) cells, mixed with viable (V) W256 cells, decreased the latent period for initiation of allogeneic tumour growth without significantly affecting its rate. This Révész effect decreased with increase in the number of inoculated V cells, and with decrease in age of recipient. LI cells of a different (Y-P388) rat tumour exerted a Révész effect, even in recipients which had been immunized with LI (Y-P388) tumour cells. Local pre-irradiation of the site of inoculation of V cells decreased both the latent period and rate of tumour growth. It acted independently of a Révész effect, and the decrease in tumour growth rate was partly due to emigration of V cells from the inoculum, producing metastases. LI, but not heat-killed cells, induced prolonged swelling of the tumour bed in unimmunized and tumour-immunized rats, which, unlike inflammatory swelling, was inhibited by pre-irradiation of the foot. It is postulated that the Révész effect is due to enhancement of survival of V cells by trophic substances which are principally elaborated by LI (AND V) cells, but also by the tumour bed, due to innate growth and trophic reactions of its tissues to the presence of tumour cells.


*Br. J. Cancer* (1977) **36**, 94

### REACTIONS OF THE TUMOUR BED TO LETHALLY IRRADIATED TUMOUR CELLS, AND THE RÉVÉSZ EFFECT

H. A. S. VAN DEN BRENK, M. C. CROWE AND M. G. STONE

*From the Richard Dimbleby Department of Cancer Research, St. Thomas's Hospital Medical School, London SE1 7EH*

Received 19 January 1977 Accepted 11 March 1977

**Summary.—Subdermal inoculation of the foot of the rat with lethally irradiated (LI) Walker tumour (W256) cells, mixed with viable (V) W256 cells, decreased the latent period for initiation of allogeneic tumour growth without significantly affecting its rate. This Révész effect decreased with increase in the number of inoculated V cells, and with decrease in age of recipient. LI cells of a different (Y-P388) rat tumour exerted a Révész effect, even in recipients which had been immunized with LI (Y-P388) tumour cells. Local pre-irradiation of the site of inoculation of V cells decreased both the latent period and rate of tumour growth. It acted independently of a Révész effect, and the decrease in tumour growth rate was partly due to emigration of V cells from the inoculum, producing metastases. LI, but not heat-killed cells, induced prolonged swelling of the tumour bed in unimmunized and tumour-immunized rats, which, unlike inflammatory swelling, was inhibited by pre-irradiation of the foot. It is postulated that the Révész effect is due to enhancement of survival of V cells by trophic substances which are principally elaborated by LI (and V) cells, but also by the tumour bed, due to innate growth and trophic reactions of its tissues to the presence of tumour cells.**

IN experimental animals the growth of transplanted allogeneic and syngeneic tumours can be markedly enhanced by adding lethally irradiated (LI) tumour cells in excess to the implanted inoculum of intact, viable (V) tumour cells. This phenomenon was discovered by Révész (1956) who showed that it depended primarily on the metabolic activities of LI cells, and their production of diffusible metabolites *in situ* which conditioned the cellular micro-environment of V cells, and established a "milieu propitieux" for their growth. Subsequent studies of the Révész effect by Hewitt, Blake and Porter (1973) based on fully quantitative transplantation bioassays of a non-immunogenic syngeneic tumour, showed that LI cells did not affect the rate of proliferation of V cells, but decreased the number of V cells required to induce tumour growth by increasing the proportion of V cells which contribute to tumour initiation. Although this effect of LI cells *in vivo* is analogous to their conditioning effect as feeder cells *in vitro* of increasing V cell-plating efficiencies (Puck and Marcus, 1955), Hewitt *et al.* (1973) considered that an interaction of LI cells with the normal tissues of the tumour bed was more important than their feeder-cell action in initiating tumour growth. Subsequently, Peters and Hewitt (1974) considered that a thromboplastic effect exerted locally by LI cells contributed to the Révész effect.

Clonogenic growth of transplanted tumour cells is enhanced in inflamed tissues (van den Brenk *et al.*, 1974). Also, tumour cells induce angioblastic reactions and vasoproliferation at the site of implantation (Folkman, 1972). The vascular nature of these reactions of the tumour bed to V and LI cells would be expected to lead to increased permeability of the microvasculature, and produce significant swelling of the tumour bed itself. In this paper we describe experiments in which changes in tissue volume were measured after the foot of the rat was subdermally (s.d.) injected with allogeneic V and LI cells, singly or in combination, by volumetric displacement, as described previously (van den Brenk *et al.*, 1977*a*). Tumour growth curves have been used to study the Révész effect, and the effects of X-irradiation of the tumour bed and tumour immunity on local reactions of the tumour bed to implanted LI cells have been measured.

### MATERIALS AND METHODS

Female Carworth Farm strain (SPF) rats were used for the transplantation of sublines of Walker (W256) and Yoshida (Y-P388) rat tumours. Techniques used for preparation of the tumours as cell suspensions for passaging and quantitative transplantation by injection, production of LI tumour cells and administration of whole body irradiation (WBI) and local irradiation have been described previously (van den Brenk, Sharpington and Orton, 1973). To prepare LI cells, freshly harvested and washed ascites tumour cells held in disposable plastic syringes were given a single dose of 14 krad X-rays. Tumour cell dilutions for cell counting and injection of rats were all made with ice-cold Tyrode's solution (pH 7·3). The volume of the rat's foot was measured by volumetric displacement as described previously (van den Brenk *et al.*, 1977*a*). V and LI tumour cells were contained in a total volume of 0·1 ml for transplantation, unless stated otherwise, and injected into the superficial s.d. connective-tissue layer of the dorsum of the right foot, an equal volume of Tyrode's solution being injected into the contralateral (left) foot. Both feet were measured throughout each experiment, but measurements were discontinued and the rats killed when ulceration and fungation of tumour appeared imminent, the tumour had grown proximal to the point of reference (level of the tip of the calcaneal tuberosity), lymph node metastases became palpable or the rat showed signs of systemic dissemination such as progressive anaemia and loss of weight. Tumour volume was calculated by subtracting the volume of the left foot from that of the right. Swelling induced by s.d. injection of the foot with LI cells or inflammatory agents and, by local X-irradiation of the foot, was measured in the same way. The effect of age of rat on its reaction to LI cells was determined. In these experiments the rate of growth of the foot injected with Tyrode's solution only was considered as normal and used to analyse the effect of the presence of tumour cells in the foot on its pattern of growth.

### RESULTS

### *Effects of admixed LI cells on growth of V cells in the foot: Influence of age of host*

Measurements made in 4- and 7-week-old rats, in which the right foot had been injected with $10^3$ W256 cells (Fig. 1), showed that tumours developed much more rapidly in the younger rats. All 7-week-old rats did eventually develop progressively growing tumours, but growth commenced in only one rat within 20 days of transplantation (see below). The addition of $2 \times 10^6$ LI (W256) tumour cells to the V cell inoculum hastened the onset of growth in both age groups, but this Révész effect was most marked in the older group. The rate of tumour growth was not significantly affected by LI cells or age of rat; all tumours grew exponentially at similar rates. A striking effect, in both young and old rats, of adding LI cells to V cells was to produce an increase in tissue volume which was sustained for the 5 to 10 days before the progressive swelling due to exponential growth of V cells supervened (Fig. 1, see below). The only significant effect of the presence of LI cells was to shorten the latent period of tumour growth. Hewitt *et al.* (1973) have attributed this effect to enhanced survival *in vivo* of the injected V cells which are responsible for tumour initiation. Our results support their view and also show that *a similarly enhanced survival of V cells occurs when the tumour bed consists of more actively growing normal tissues in young animals.*

The latent period was shortened, but growth rate not significantly affected,

FIG. 1.—Changes in volume of foot of individual rats produced by s.d. injection of $10^4$ V (W256) cells (closed circles) or $10^4$ V (W256) *plus* $2 \times 10^6$ LI (W256) cells (open circles) suspended in 0·1 ml Tyrode's solution. Contralateral foot was injected with 0·1 ml Tyrode's solution (dots; interrupted lines); left graphs: 4-week-old rats; right graphs: 7-week-old rats.

when LI cells were mixed with 10 to $10^4$ V cells for injection; this shortening of the latent period increased with decrease in number of transplanted V cells (Fig. 2). This suggests that, as the population density of V cells at the site of inoculation increases, the proportion of V cells which survive and initiate tumour growth increases. It follows that a conditioning (Révész) effect is autonomously generated by V cells, which is population-density dependent. Consequently, transplanted V cells become proportionately less dependent on the presence of added LI cells for their survival and growth as their population density in the inoculum increases. The latent period of tumour growth was also significantly shortened by only injecting V cells suspended in freshly harvested, heparinized rat pleural fluid, but the latter was less effective than LI cells in this respect. About 50% of tumours produced by smaller V cell inocula (10 to $10^3$ cells) regressed rapidly

FIG. 2.—Tumour growth curves in 9 groups of 4-week-old rats (6 rats per group) produced by V (W256) cells (closed symbols) or V (W256) *plus* $2 \times 10^6$ LI (W256) cells (open symbols). Number of inoculated V cells: 10 (▼ ▽), $10^2$ (■ □), $10^3$ (▲ △ ×), $10^4$ (● ○). In one group (×) the V cells were suspended in 0·1 ml rat pleural fluid for s.d. injection; in the other groups, V or V plus LI cells were suspended in Tyrode's solution. Contralateral feet were injected with 0·1 ml Tyrode's solution.

FIG. 3.—Tumour growth curves in two groups of 10-week-old rats produced by s.d. injection of foot with $10^2$ V (W256) cells (closed circles) or $10^2$ V *plus* $2 \times 10^6$ LI (W256) cells (open circles) (5 rats per group).

FIG. 4.—Tumour growth curves in unirradiated rats (closed symbols) and rats given 570 rad whole-body irradiation (open symbols), produced by s.d. injection of foot with $10^5$ (■ □), $3{\cdot}3 \times 10^5$ (○ ●) and $10^6$ (▲ △) V (W256) cells; 7-week-old rats, 4 rats per group.

and completely after having grown exponentially for 3 to 5 days (Figs. 1, 2, 3). Such regression was invariably sudden in onset and continued at near-exponential rates (see Fig. 1). LI cells added in excess to V cells reduced the incidence but not the pattern or rate of regression, even if the latent period was increased to 14 days or more by injecting older rats with fewer V cells (Fig. 3). It follows that any tumour immunity which was induced by injecting older rats with a single large dose of allogeneic and immunogenic tumour LI cells, was much less significant than the Révész effect, in influencing the survival and growth of a small homologous V cell inoculum. The acute tissue swelling caused by injecting the foot with LI cells persisted for more than a week in 10-week-old rats (Fig. 3). The intensity of this reaction did not appear to be significantly affected by age of host.

*Effects of tumour immunity on tumour growth*

(*i*) *Immunosuppression.* — Sublethal WBI, given to suppress the development of tumour immunity in rats, had no significant effect on either the latent period or initial rate of tumour growth, but appeared to precipitate premature arrest of growth in the foot of smaller V cell inocula (Fig. 4). During this arrest of local growth, however, the incidence of metastases in the regional popliteal and lower abdominal lymph nodes greatly increased. It follows that the arrest of local growth in irradiated rats is partly due at least to excessive *loss* of viable tumour cells from the site of inoculation, which enter lymphatics and grow elsewhere in the animal. Admixture of V cells with LI cells did not decrease lymphatic dissemination after WBI (or local irradiation of the foot; see below). This effect of WBI, of arresting local growth and enhancing dissemination, resembled the effect produced in unirradiated rats by injecting the tumour cells deeply into the loose intertendinous tissues of the foot (van den Brenk *et al.*, 1977*a*, see below).

(*ii*) *Immunization.*—S.c. injection of rats with $2 \times 10^6$ or more allogeneic LI cells twice weekly for 3 weeks induced tumour-specific immunity and increased the number of intramuscularly (i.m.) injected W256 or Y-P388 tumour V cells required to induce progressive growth of tumour in 50% of rats ($TD_{50}$ value) from

$<10^2$ cells to $>10^5$ cells (van den Brenk, Moore and Sharpington, 1971). Similar pretreatment of 7-week-old rats, with $5 \times 10^6$ LI (W256) cells injected twice weekly for only 2 weeks, failed to significantly affect the times of onset and rates of growth of $10^5$–$10^6$ s.d. injected V (W256) tumour cells in the foot (results not tabulated). Prolonged pretreatment of rats with LI cells for several weeks was required to prevent s.d. growth in the foot of such larger inocula (see below).

*Tissue swelling induced by LI cells.*—S.d. injection with $10^6$ or more LI tumour cells invariably induced an immediate swelling of the foot which approximated in volume to that of the injection and persisted for several days before it gradually subsided. This LI-cell-induced swelling similarly occurred when V cells were mixed with LI cells (see Figs 1 and 3). It was not produced by injecting saline, serum, plasma, pleural fluid or heat-killed tumour cells (treatments which caused transient swellings which disappeared within minutes of injection). Injected LI cells did not induce significant

FIG. 5.—Mean changes in body weight and volume of feet of 2 groups of 3 rats in which one foot was injected s.d. with 0·1 ml Tyrode's solution (dots, small closed triangles; solid line) or with $5 \times 10^6$ LI (W256) cells suspended in 0·1 ml Tyrode's solution (open symbols, interrupted lines), at 30 days (circles) or 46 days (triangles) of age.

reddening, warmth or oedema of the foot, thus differing from an exudative (inflammatory) reaction to tissue injury, in which the increase in tissue volume greatly exceeds that due to tissue displacement by the injurious material responsible for the inflammatory reaction. In younger rats, with more rapidly growing feet, the swelling caused by injected LI cells was progressively "taken up" by postnatal growth of the foot (Fig. 5); *i.e.* the increases in volume, caused by normal growth and by LI cells respectively, were not additive but appeared to be complementary in maintaining allometric (proportionate) growth of the foot. Consequently, the tissue swelling induced by LI cells appeared to be of shorter duration in younger rats than in older ones.

*Effect of local irradiation of foot on swellings induced by inflammatory agents and LI cells*

The acute exudative reactions of the foot to injection with injurious agents and endogenous mediators of the inflammatory response to injury (namely histamine, 5-hydroxytryptamine, bradykinin and prostaglandin) were found to be highly radioresistant (to be published). Thus, local pre-irradiation of the foot with a single dose of 20 krad had no significant effect on the intensity or resolution of the swellings induced by these agents. The acute exudative (inflammatory) reaction induced by agents, (such as Compound 48/80) which release pharmacologically active endogenous mediators of inflammation from depots in tissues, was similarly radioresistant in both young and old rats. These results confirm previously reported experimental findings concerning the radioresistance of acute inflammatory reactions in the skin (van den Brenk, 1958).

The reaction of tissues to implanted LI tumour cells differed from an inflammatory reaction in being inhibited by local X-irradiation. Pre-irradiation of the foot of 7-week-old rats with a single dose of 1500–2000 rad abolished the swelling induced by LI cells, without significantly reducing the rate of growth of the foot over the period of observation (Fig. 6).

*Effect of implantation of LI and V cells in tumour-immunized hosts*

In rats which had been immunized against growth of W256 cells by s.c. injections of LI (W256) cells into the nape of the neck, given twice weekly for several weeks, the intensity and duration of the swelling induced by s.d. injection of the foot with LI cells was not significantly altered (Fig. 7). Two further injections into the foot of these

FIG. 6.—Changes in volume of right foot of 7-week-old rats injected with LI (W256) cells in suspension (open symbols) and of contralateral foot injected with equal volume Tyrode's solution (closed symbols). A and E. 2 × 10⁶ LI cells (0·1 ml); B. 4 × 10⁶ LI cells (0·2 ml); C and F. 2 × 10⁶ heat-killed (60°C for 30 min) LI cells; D. 4 × 10⁶ heat-killed LI cells. In E and F the right foot of each rat was locally irradiated with 2000 rad X-rays 10 min before injection. Four rats per group.

FIG. 7.—Effect on volume of right foot of s.d. injection of foot in W256 tumour-immunized rats with lethally irradiated (*LI*) W256 cells (5 × 10⁷/ml) and unirradiated (*L*) W256 cells (10⁷/ml). The rats were injected s.c. (nape of neck) twice weekly for 4 weeks with 0·5 × 10⁶ or 10⁷ LI (W256) cells before injecting the foot. Plotted points show mean values; vertical bars show maximum and minimum values, for 3 rats.

immunized rats with 10⁶ and 2 × 10⁶ V cells caused swellings of similar duration which resolved without growth of tumour (Fig. 7). Local pre-irradiation of the tumour bed in the foot in tumour-immune rats abolished the swelling induced by injecting LI or V cells (see below).

*Local pre-irradiation of the foot: Effects on tumour growth and Révész effect*

Local irradiation of the foot with a single dose of 3000 rad caused no significant change in tissue volume for 10 days, when erythema and dry desquamation of the skin, associated with a very modest increase in volume of the foot, occurred. The latter subsided after a week, when the skin reactions resolved. Increasing the dose to 5000 rad caused the foot to swell significantly within 24 h. This swelling subsided in 2 days, but a recurrent swelling appeared on the fifth day, which progressively increased as the foot developed severe erythema, followed by moist desquamation and patchy superficial necrosis of the epidermis. At about 14 days after irradiation, this swelling had reached a maximum intensity and did not show any decrease for a further week. These changes induced by local irradiation

FIG. 8.—Changes in volume of foot produced by its local irradiation with a single dose of 3000 rad (+) or 5000 rad (×) and produced by growth of $10^3$ V (W256) cells injected s.d. into unirradiated foot (●) or immediately after irradiation of foot with 3000 rad (○). Six 8-week-old rats per group.

of the foot are shown in Fig. 8, together with the effect produced by pre-irradiation (3000 rad) of the tumour bed on the growth of a small inoculum of $10^3$ V (W256) cells. It is seen that pre-irradiation of the tumour bed greatly increased the latent period for tumour growth, but did not decrease its rate. Similar measurements in pre-irradiated feet were made when the number of inoculated V cells was increased to $10^5$ cells, and when LI cells were mixed with V cells for injection (Fig. 9). Pre-irradiation of the foot inhibited the swelling induced by LI cells. It did not significantly alter shortening of the latent period for growth induced by LI cells (*i.e.* Révész effect) but caused marked decreases in the rates of tumour growth 10 to 15 days after implantation, irrespective of whether LI cells had been added to the inoculum. However, these decreases in tumour growth rate caused by pre-irradiation of the foot were accompanied by the appearance and rapid growth of the regional lymph node metastases, and are attributed to the accelerated loss of *viable* tumour cells from the site of inoculation and their lymphatic dissemination. A similar effect was observed following WBI (Fig. 3; see above).

FIG. 9.—Increase in volume of foot produced by s.d. injection of $10^5$ V (W256) cells (closed symbols) or $10^5$ V (W256) *plus* 2 × $10^6$ LI (W256) cells (open symbols) in unirradiated feet (circles) and in feet pre-irradiated with 2000 rad X-rays immediately before injection (triangles). Five or six 10-week-old rats per group.

*Influence of interval between injection of LI cells and V cells on Révész effect*

LI cells caused the greatest reduction in latent period of tumour initiation when LI cells and V cells were implanted at the same time; the single injection of LI cells mixed with V cells was no more effective in this respect than the injection of LI and V cells separately into the same tissue locus. Injection of LI cells separately, one or more days after V cells, reduced the Révész effect (Fig. 10) as described previously by Hewitt *et al.* (1973) but did not abolish it altogether when fewer ($10^3$) V cells were inoculated, which increased the latent period for initiation of growth to 2 weeks or more. Indeed, LI cells injected as long as 4 days after $10^3$ V cells, significantly reduced the latent period, which suggests that their action is concerned not only with enhancing the survival of inoculated V cells but also that of V cell progeny. Loss of the latter from the inoculum can apparently occur at an early (cryptic) stage of tumour development when the population density of tumour cells remains low. During this phase, V cells in the inoculum continue to be susceptible to a Révész

FIG. 10.—Effect of separately injecting $2 \times 10^6$ LI (W256) cells, immediately (○), 1 day (□) or 4 days (△) after injecting $10^3$ V (W256) cells into the same s.d. locus in the foot, compared with rats in which $10^3$ V cells only were injected (●). Four 20-week-old rats per group.

effect exerted by artificially incorporated LI cells, and conceivably also by LI cells which form when the tumour is locally irradiated. In higher-density V cell inocula, however, the Révész effect due to LI cells becomes less relevant, since this conditioning effect is being autonomously generated by V cells in proportion to their population density (see below).

*Tumour immunity and Révész effect*

Rats were immunized against either W256 or Y-P388 tumour, by s.c. injecting rats with $10^6$–$10^7$ LI cells of the corresponding tumour twice weekly for not less than 4 weeks. In unimmunized rats, LI (Y-P388) cells exerted a Révész effect against an inoculum of $10^3$ V (W256) cells by shortening the latent period for tumour growth. In W256-immunized rats no tumours grew when $10^3$ V (W256) cells were injected into the foot, even if $10^7$ LI (W256) were mixed and injected with the V cells. In Y-P388-immunized rats however, $10^3$ V (W256) cells caused tumour growth, and the latent period was significantly shortened by addition of either $10^7$ LI (W256) or $10^7$ LI (Y-P388) cells to the V (W256) inoculum (results not tabulated).

DISCUSSION

The original studies of Révész (1958) established that locally acting diffusible products of LI cell metabolism *in vivo* were fundamentally involved in the enhancement of growth of V tumour cells by admixed LI cells. He postulated that LI cells probably acted as feeder cells, by exerting a conditioning action similar to that of increasing plating efficiency (clonogenic growth) of cultured cells *in vitro* (Puck and Marcus, 1955). Révész nevertheless considered the possibility that the LI-cell effect was mediated by their actions on the normal tissues (tumour bed) into which the cells had been inoculated, and that reactions of an inflammatory nature were involved. In their studies of the Révész effect, Hewitt *et al.* (1973), using fully quantitative isogeneic tumour transplantation assays, clearly demonstrated that LI cells acted *in vivo* by "increasing the proportion of viable cells which contribute to tumour initiation", and reported that "there was no evidence that LI cells affected the rate of proliferation of viable cells". Our own measurements of s.d. growth by volumetric displacement, of allogeneic tumour cells implanted in the foot of the rat, have fully supported their findings in this respect. We are more reluctant than Hewitt *et al.* (1973) however, to rule out the view that a "feeder cell" action is primarily involved, directly nurturing and increasing the survival of V cells in the inoculum, and thereby enhancing initiation of tumour growth. The conditioning effect of LI cells is not simply concerned with elaboration of relatively non-specific nutrients which are present in abundance in artificial culture media and tissue fluids. We support the view that LI or other feeder cells elaborate compounds which are essentially trophic hormones (specific substances with short metabolic lives *in vivo*)

which nurture V cells. These factors are likewise elaborated by V cells and are present in serum and tissue fluids. They enhance survival, and thereby maintain the potential for replicative growth of V cells, by conditioning the microenvironment. Since inoculated LI cells can survive for several days *in vivo* as non-proliferating but otherwise metabolically active cells, they exert a much more sustained feeder-cell effect on inoculated V cells than conditioning factors, present in sera or other tissue fluids, added to the inoculum. Such additives rapidly disappear as the result of diffusion and metabolic degradation, and cannot sustain the trophic action. The auto-conditioning effect produced by V cells themselves determines that any enhancement of tumour initiation produced by added LI cells (Révész effect) is greatest when inocula consist of very few V cells, and decreases as the inoculum cell density increases. This disadvantage suffered by small V-cell inocula in maintaining concentrations of growth factor in their micro-environment which are required to ensure cell survival and growth is clearly greatest when single cells are inoculated and each cell is required to clone for proliferative growth to be initiated. The survival and clonogenic growth of i.v.-injected tumour cells in the lungs of rats are greatly increased by simultaneous injection of LI cells, if the recipient animal is young (recently weaned) and actively growing, or if reactive hyperplasia of target tissues has been induced by injurious stressor agents, irrespective of age of rat (van den Brenk *et al.*, 1974). We have attributed these effects of age and tissue stress on tumour colony-forming efficiency (CFE) to enhanced local elaboration of trophic agents by the tumour bed in states of rapid innate or reactive growth; an effect analogous to the Révész effect produced by LI cells, which actively growing normal tissue exercises. This view is supported by the finding that in the absence of LI cells, tumour growth is initiated by small V cell inocula much more rapidly in the foot of weanling rats than in older rats (Fig. 1).

LI cells derived from transformed aneuploid cell lines have proved most effective in exerting a feeder-cell effect *in vitro*, or a Révész effect *in vivo*. Such cells also survive far longer than normal cells after sterilization of cell replicative integrity by X-rays. After irradiation, their inherent capacity for continuous growth is manifested by hypertrophic growth, and the frequent resumption of DNA synthesis; aberrant division causes polyploidy and multinuclearity, and a population of "radiation giant" cells survives. It is significant that the exertion of a Révész effect by cells seems to be largely governed by their capacity to survive *in vivo* and sustain metabolic activities specifically associated with growth. Whereas unirradiated or irradiated normal adult tissue cells have rarely been reported to exert this effect, Schneyer (1955) found that normal embryo cells, mixed with tumour cells, caused stimulation of tumour growth.

The possibility that LI cells induce changes in normal tissues which protect the tumour-cell implant has been reinvestigated by Peters and Hewitt (1974). They concluded that LI cells induced a thromboplastic effect in the tumour bed, which supported the tumour cells and was presumably vascular in origin. They found that mixture of V cells with certain tissue homogenates and erythrocytes which cause fibrin to form at the site of injection, simulated LI cells in producing a Révész-like effect. They postulated that fibrin produced a barrier which prevented the escape, apparently by migration, of V cells from the site of implantation. The escape of V cells in the absence of such a barrier presumably resulted in death of the emigrant V cells in adjacent tissues, but the cause of their death has not been clarified or confirmed. In our view, a demise of emigrant cells could be equally well attributed to their escape from the conditioning effect of tumour-cell-derived trophic substances, which "pool" in the micro-environment of a high-cell-density parent-tumour-cell inoculum. It is cogent also, that many tissue homogenates and preparations of fibrinogen cause inflammation, which has been shown to be conducive to survival, take and clonogenic growth of seeded tumour cells, even in animals treated with anti-coagulant drugs in high dosages to inhibit blood clotting (van den Brenk *et al.*, 1974). However, we have shown that the swelling of the foot caused by LI cells differs in important respects from the typical acute exudative phase of an inflammatory reaction to an injurious agent. Thus, LI cells do not cause the excessive leakage of plasma from capillaries into the tissues associated with inflammatory states. They produce a swelling caused by displacement, which is sustained for several days before it slowly abates, and which is progressively "taken up" by the increase in normal tissue volume produced by natural growth. Unlike inflammatory swelling, LI-induced swelling is radiosensitive. The tissue swelling and Révész effect induced by LI cells, depend on their capacity to continue and sustain their metabolic activities in the tissues; thus, heat-killed LI (or V) cells cause no significant swelling or Révész effect (Révész, 1958; Fig. 8) in either unirradiated or irradiated tissues. V tumour cells induce rapid angiogenesis (Algire and Chalkley, 1945; Folkman *et al.*, 1971). Angiogenesis, which is similarly induced by LI cells, is abolished by local X-irradiation of the tumour bed (van den Brenk *et al.*, 1977*b*). These findings suggest that the rapid induction of blastogenic, followed by mitogenic, changes in the tumour bed by trophic factors released by LI (and V) cells may be indirectly involved in the Révész effect. The concept that succour, survival and growth of implanted tumour cells depend on "milieu propitieux" at the site of inoculation or seeding, to which blastogenesis and growth of the tumour bed contribute, is supported by the considerable influence which age of recipient animal (and the innate rate of growth of the tumour bed) exercises on initiation of tumour growth (Fig. 1; van den Brenk *et al.*, 1973).

Pre-irradiation of the tumour bed decreased the rate of tumour growth without apparently altering the $TD_{50}$ for tumour induction (Hewitt and Blake, 1968). This so-called "tumour bed effect" of irradiation occurs independently of the Révész effect (Fig. 12) and the two effects clearly differ in their mechanism of action. We postulate that the tumour-bed effect may be primarily due to a breakdown by X-rays of physiological tissue barriers which otherwise help to constrain emigration of tumour cells from their site of origin. Thus, the rapid cellular depopulation induced by X-rays in epithelial and other dividing tissues would lead to breakdown of basement membranes and similar endoskeletal frameworks. This would provide access of tumour cells to neighbouring regions and facilitate their entry into lymphatic spaces. Alternatively, the possibility exists that the tumour-bed effect is due to irradiation atrophy and devascularization of normal tissues, which interfere with metabolism and growth of the tumour. Clearly, further studies are required to elucidate the interactions between the tumour and tumour bed, which have major implications in radiotherapy and tumour radiobiology.

Our studies clearly support the findings of Révész (1956, 1958) that a strong Révész effect is exerted by highly immunogenic allogeneic tumours. We have shown that, if significant tumour immunity is raised against V cells by presence in the inoculum of a large population of homologous immunogenic LI cells, it fails to compete with the Révész effect that the latter exert, and does not significantly affect the growth of a primary challenge of rapidly growing anaplastic cells of a tumour such as the Walker carcinoma. Indeed, despite their immunogenicity, the presence of LI cells in the inoculum reduced the number of inoculated V cells required to initiate tumour growth, even when fewer V cells were inoculated to lengthen the latent periods of growth and thereby facilitate the development of immunity. Indeed, immunity specifically induced in rats to destroy LI cells which differed in their antigenic determinants from V cells used to induce tumour growth, did not prevent such vulnerable LI cells from exerting the Révész effect. It follows that growth in the animal of rapidly growing, allogeneic tumour cells, despite their immunogenicity, is less influenced by immunological incompatibility than by trophic factors. The latter depend on auto-conditioning of the inoculum, which increases with V-cell density the presence of LI cells (Révész effect), animal age and the innate rate of growth of the tumour bed, and perturbed growth of the tumour bed induced by stressors and growth promoting agents. Immunological reactions take time to develop, even in animals in which LI tumour cells in large numbers have been injected over a period of weeks to induce immunity. During this period of immunological inactivity, antigenic LI cells enhance tumour growth by inducing a Révész effect, which may be misinterpreted as an immunological phenomenon. Similarly, situations exist in which whole-body and local irradiation promote tumour growth, which are commonly explained in terms of immuno-suppression, and may be offered as indirect evidence of tumour immunogenicity even when a syngeneic or autochthonous tumour–host relationship prevails. The possibility, however, that stimulation of tumour growth under such conditions results from trophic effects induced by X-rays in normal tissues should not be overlooked.

### REFERENCES

ALGIRE, G. H. & CHALKLEY, H. W. (1945) Vascular Reactions of Normal and Malignant Tissues *In Vivo*. I. Vascular Reactions of Mice to Wounds and to Normal and Neoplastic Implants. *J. natn. Cancer Inst.*, **6**, 73.

FOLKMAN, J., MERLER, E., ABERNETHY, C. & WILLIAMS, G. (1971) Isolation of a Tumor Factor Responsible for Angiogenesis. *J. exp. Med.*, **133**, 275.

FOLKMAN, J. (1972) Anti-angiogenesis: New Concept for Therapy of Solid Tumours. *Ann. Surg.*, **175**, 409.

HEWITT, H. B. & BLAKE, E. R. (1968) The Growth of Transplanted Murine Tumours in Pre-irradiated Sites. *Br. J. Cancer*, **22**, 808.

HEWITT, H. B., BLAKE, E. R. & PORTER, E. H. (1973) The Effect of Lethally Irradiated Cells on the Transplantability of Murine Tumours. *Br. J. Cancer*, **28**, 123.

PETERS, L. J. & HEWITT, H. B. (1974) The Influence of Fibrin Formation on the Transplantability of Murine Tumour Cells: Implications for the Mechanism of the Révész Effect. *Br. J. Cancer*, **29**, 279.

PUCK, T. T. & MARCUS, P. I. (1955) A Rapid Method for Viable Cell Titration and Clone Production with HeLa Cells in Tissue Culture: The Use of X-irradiated Cells to Provide Conditioning Factors. *Proc. natn. Acad. Sci., U.S.A.*, **41**, 432.

RÉVÉSZ, L. (1956) Effect of Tumour Cells Killed by X-rays Upon the Growth of Admixed Viable Cells. *Nature, Lond.*, **178**, 1391.

RÉVÉSZ, L. (1958) Effect of Lethally Damaged Tumor Cells upon the Development of Admixed Viable Cells. *J. natn. Cancer Inst.*, **20**, 1157.

SCHNEYER, C. A. (1955) Effect of Normal Tissue Inocula on Homologous Tumor Transplants. *Cancer Res.*, **15**, 268.

VAN DEN BRENK, H. A. S. (1958) Observations on Mast Cell Changes, Histamine Release and Local Tissue Damage in Rats Following X-irradiation. *Br. J. exp. Path.*, **39**, 356.

VAN DEN BRENK, H. A. S., MOORE, V. & SHARPINGTON, C. (1971) Growth of Metastases from P388 Sarcoma in the Rat Following Whole Body Irradiation. *Br. J. Cancer*, **25**, 186.

VAN DEN BRENK, H. A. S., SHARPINGTON, C. & ORTON, C. (1973) Macrocolony Assays in the Rat of Allogeneic Y-P388 and W-256 Tumour Cells Injected Intravenously: Dependence of Colony Forming Efficiency on Age of Host and Immunity. *Br. J. Cancer*, **27**, 134.

VAN DEN BRENK, H. A. S., STONE, M., KELLY, H., ORTON, C. & SHARPINGTON, C. (1974) Promotion of Growth of Tumour Cells in Acutely Inflamed Tissues. *Br. J. Cancer*, **30**, 246.

VAN DEN BRENK, H. A. S., STONE, M., BURNS, J. W. & CROWE, M. C. (1977a) Rapid and Accurate Measurement of Growth of Solid Tumours and Changes in the Tumour Bed in the Rat by the Technique of Volumetric Displacement. *Br. J. Cancer*, **35**, 92.

VAN DEN BRENK, H. A. S., CROWE, M., KELLY, H. & STONE, M. G. (1977*b*) The Significance of Free Blood in Liquid and Solid Tumours. *Br. J. exp. Path.*, **58**, 147.